# Uncovering the key working mechanisms of a complex community-based obesity prevention programme in the Netherlands using ripple effects mapping

**DOI:** 10.1186/s12961-024-01182-y

**Published:** 2024-09-04

**Authors:** Irma Huiberts, Dorine Collard, Amika Singh, Mara Hendriks, Mai J. M. Chinapaw

**Affiliations:** 1 Amsterdam UMC, Location Vrije Universiteit Amsterdam, Public and Occupational Health, De Boelelaan 1117, 1081HV Amsterdam, The Netherlands; 2 Mulier Instituut, Herculesplein 269, 3584AA Utrecht, The Netherlands; 3 Amsterdam Public Health, Health Behaviors & Chronic Diseases and Methodology, Amsterdam, The Netherlands; 4 Human Movement, School and Sport, Applied University of Windesheim, Campus 2, 8017CA Zwolle, The Netherlands

**Keywords:** Health promotion, Obesity, Community based, Prevention, Child, Complexity, Mechanisms, Healthy lifestyle, Stakeholders, Implementation, Intersectoral

## Abstract

**Background:**

Much remains unknown about how complex community-based programmes can successfully achieve long-term impact. More insight is needed to understand the key mechanisms through which these programmes work. Therefore, we conducted an in-depth study in five communities that implemented the Healthy Youth, Healthy Future (JOGG) approach, a Dutch community-based obesity prevention programme. We aimed to identify perceived outcomes and long-term impacts among local stakeholders and explore potential causal pathways and working mechanisms.

**Methods:**

We used ripple effects mapping (REM), a qualitative participatory method to map outcomes and identify causal pathways, in five communities. We involved 26 stakeholders, professionals and policy-makers affiliated with the local JOGG approach, spread over eight REM sessions and conducted individual interviews with 24 additional stakeholders. To uncover working mechanisms, we compared outcomes and causal pathways across communities.

**Results:**

Over 5–9 years of implementation, participants perceived that JOGG had improved ownership of local stakeholders, health policies, intersectoral collaboration and social norms towards promoting healthy lifestyles. Causal pathways comprised small initial outcomes that created the preconditions to enable the achievement of long-term impact. Although exact JOGG actions varied widely between communities, we identified five common working mechanisms through which the JOGG approach contributed to causal pathways: (1) creating a positive connotation with JOGG, (2) mobilizing stakeholders to participate in the JOGG approach, (3) facilitating projects to promote knowledge and awareness among stakeholders while creating successful experiences with promoting healthy lifestyles, (4) connecting stakeholders, thereby stimulating intersectoral collaboration and (5) sharing stakeholder successes that promote healthy lifestyles, which gradually created a social norm of participation.

**Conclusions:**

The JOGG approach seems to work through activating initial stakeholder participation and bolstering the process towards ownership, policy change, and intersectoral collaboration to promote healthy lifestyles. Key working mechanisms can inform further development of JOGG as well as other complex community-based prevention programmes.

**Supplementary Information:**

The online version contains supplementary material available at 10.1186/s12961-024-01182-y.

## Background

Childhood overweight and obesity are a worldwide health concern [1, 2] with vast consequences for children’s health and well-being[3–6], as well as population health and the economy[1, 7]. Implementing community-based obesity prevention programmes has been an important strategy for overweight prevention in the population [7–9]. These programmes address the environmental drivers of unhealthy behaviours by involving community stakeholders in the development and implementation of actions that fit the local situation.

In the Netherlands, a widely implemented community-based programme is the Healthy Youth, Healthy Future (JOGG) approach, which is currently active in more than two thirds of the municipalities in the Netherlands. JOGG aims to promote a healthy lifestyle among children and youth (0–21 years old) as well as prevent obesity by creating a health-promoting environment in different settings (school, childcare, sport clubs and neighbourhoods). To do this, JOGG employs a community capacity-building approach, creating awareness and shared ownership of the problem while supporting local stakeholders’ actions [10–13]. To foster the transformation towards a health-promoting environment, JOGG implementers continuously adapt their strategy to the changing local context, the response of stakeholders, and emergent outcomes [10, 14]. Successful implementation is therefore considered to vary between contexts and over time.

Much remains unknown about the working mechanisms of complex community-based obesity prevention programmes, such as the JOGG approach. Although some key elements and principles for best practices have been identified [11, 13, 15], few scholars have extensively investigated the complex process from implementation to outcome and long-term impact [16–20]. Process evaluations of community-based programmes to date have focused on measuring implementation (such as exposure and fidelity) or changes in predefined outcomes (such as environmental changes) [17] and therefore provide limited insight into how the programmes produce outcomes [21]. Additionally, most researchers have not considered complexity of the implementation of community-based programmes: the unintended and emergent outcomes [22], long-term impact, the complex causal chain through which the programme interacts with the context [17, 24, 25] and how outcomes, impact and successful implementation strategies consequently vary between contexts and evolve over time [14, 23].

Gaining insight into the complex causal chain through which community-based programmes contribute to outcomes and impact could significantly improve the understanding of how they work [17, 21] and how lasting results are achieved. This involves examining which outcomes emerge over time—including those that extend beyond intended programme outcomes—and studying the interactive implementation process through which different programme elements, contextual elements and outcomes eventually lead to long-term impact [10, 14, 24, 25]. Since local stakeholders play a key role in community-based programmes, involving them is essential to gain a better understanding of how the programme works [26].

A promising method for identifying emergent outcomes, long-term impact, and the series of events through which these are accomplished is ripple effects mapping (REM) [27–29]. REM is a qualitative participatory method that engages stakeholders who are involved in or affected by the programme in mapping the intended and unintended outcomes and impacts or ripples. Instead of attributing outcomes to the programme, REM focuses on the contribution of programme implementation to outcomes and impacts within the context. Consequently, applying REM can elucidate how outcomes on different levels and settings are related [30] and how combined efforts (from the programme as well as other initiatives in the community) work synergistically to support long-term impact [27].

Therefore, in this study, we applied REM to identify the outcomes and long-term impacts of the JOGG approach as perceived by local stakeholders and to explore potential causal pathways to these outcomes and impact. By gaining insights into the causal pathways in different contexts, we can identify the key working mechanisms of the JOGG approach, which can inform further development of the JOGG approach and other community-based obesity prevention programmes [10].

## Methods

We applied a qualitative study design based on REM guidelines [27, 28]. We conducted this study in five JOGG communities, which we defined as an area where the JOGG approach has been implemented, usually one or more neighbourhoods in a municipality in the Netherlands. A local JOGG team, which comprises a policy officer and programme coordinator, is responsible for implementation. Further details regarding methodology are available in Supplementary file 1.

### Participants

Potential JOGG communities for this study were randomly selected from a list of those where the JOGG approach had been implemented for at least 5 years prior to our study (*n* = 89). We purposively sampled for maximum variations in population size, the coordinator’s employing organization and working hours per week appointed for the JOGG approach. These characteristics have previously been identified as relevant factors for variations between communities [31, 32]. Previously we conducted case studies in nine communities (for more detail, see [33]); of these, we invited the six communities that implemented JOGG as a distinct programme to participate in REM. One community declined because of a lack of time and staff turnover. We chose to exclude the three communities that integrated JOGG fully into another programme because the focus of the programme in these communities had undergone substantial changes over the years, and the implementation of JOGG as such and use of the JOGG name had diminished [33]. Therefore, gaining insight in JOGG outcomes and causal pathways would have been more difficult and required a different approach. Table 1 presents the characteristics of the five participating communities.

**Table 1:** Characteristics of participating JOGG communities

Case	Start year of JOGG in the community	Population size of JOGG community^a^	Implementing organization^b^	Hours appointed for JOGG coordination (per week)	Number of participants
1	2016	Small	Public health service	24	REM session 1: *n* = 5 REM session 2: *n* = 4 Additional interviews: *n* = 2
2	2012	Medium	Social welfare organization	32	REM session 1: *n* = 3 REM session 2: *n* = 3 Additional interviews: *n* = 8
3	2014	Large	Municipal government	28	REM session 1: *n* = 3 Additional interviews *n* = 4
4	2012	Medium	Sport service organization	20	REM session 1: *n* = 2 REM session 2: *n* = 3 Additional interviews *n* = 5
5	2015	Small	Sport service organization	0	REM session 1: *n* = 3 Additional interviews *n* = 5

^a^Small (< 50 000 residents), medium (50 000–100 000 residents), large (100 000–170 000 residents)

^b^Municipal government or public organizations

For each community, we composed a list of organizations or persons who had been involved in the JOGG approach, through a local JOGG partnership or collaboration with the local JOGG team in the past, using existing programme plans and suggestions from the local JOGG team. These stakeholders were invited to participate in a REM session. Table 1 also presents the number of participants per session in each community. Participants included professionals from schools, childcare, youth work, health service organizations, sport service organizations, private organizations (for example, supermarket) and policy-makers. After each REM session, we asked participants to suggest other stakeholders whom they considered relevant to improve our understanding of the JOGG implementation process in their community. Suggested stakeholders were then invited to participate in an additional individual interview. In total, 54 people were invited to participate in either an REM session or interview; four declined participation due to a lack of time.

### Data collection

All data were collected between February and August 2022. We conducted eight REM sessions with 26 participants in total and 24 additional individual interviews. All REM sessions and interviews were conducted online, initially because of the coronavirus disease (COVID-19) regulations and later to maintain consistency in research methods as well as to accommodate participants’ schedules. We performed the REM sessions in small groups to facilitate online discussion, and we aimed to include between three and six participants in each session, which resulted in one or two sessions in each community.

REM sessions lasted between 75 and 90 min and two facilitators were involved in each session (IH and MH). Additional interviews were conducted by one interviewer (either IH or MH) and lasted approximately 30 minutes. All sessions and interviews were audio recorded and transcribed verbatim. Informed consent was collected from all participants before the REM session or interview. Protocols for the REM sessions and interviews can be found in Supplementary file 2.

#### Ripple effects mapping sessions

We developed the protocol based on REM guidelines [27, 28], primarily following the theming and rippling method described by Chazdon et al. (2017). IH facilitated the discussions during all REM sessions. Meanwhile, MH mapped discussions onto the online whiteboard tool Miro [34]. The whiteboard was visible for participants and we aimed for the mapping process to be a joint exercise. Therefore, participants were asked to review how their comments showed up on the map and share their suggestions for changing the map if it did not reflect what they said. In addition, the facilitators asked clarifying questions to verify how the map could illustrate the discussions.

The four steps in each session began with discussions regarding the outcomes of which participants were most proud (appreciative inquiry) and were most important from the JOGG approach in the community. Second, facilitators mapped the outcomes onto the online whiteboard and prompted participants to add other outcomes [34]. The map distinguished direct outcomes, indirect short-term outcomes and indirect long-term outcomes. Participants were also prompted to consider unintended or negative consequences. Actions undertaken by the JOGG team were added to the map when mentioned by participants. Third, facilitators and participants discussed the relationships between outcomes and the ripple effects of these outcomes. Finally, facilitators asked the participants to reflect on the most interesting facets of the map, noting any missing elements and possible improvements as well as next steps in the local JOGG approach.

Each REM session resulted in a complete map. After each session, facilitators tidied the map and checked it against the audio recordings to ensure the map accurately described the discussions throughout the session. In the communities in which we conducted two REM sessions we combined the two maps. New information from the second map was incorporated into the first map. We clustered similar outcomes and processes, while retaining the original wording from both maps. After all REM sessions in the community, the (combined) map was sent to the participants to verify whether the map adequately illustrated their ideas. We directly incorporated the minor adaptations that we received from participants into the map.

We refined the REM protocol after each session. For example, we noticed significant variations in the extent to which participants were involved in the JOGG approach. This prompted us to add further differentiation in the interview protocol. For those participants who were substantially involved in the JOGG approach, we followed the theming and rippling method to gain insight into the broadness of all JOGG outcomes. For those participants who had been involved in the JOGG approach to a limited extent (that is, in one small project), we assumed an in-depth approach, asking them to share their stories and outcomes to create more detailed descriptions.

#### Additional interviews

As suggested by Chazdon et al. [27], we conducted additional individual interviews with stakeholders who were not present in the initial REM sessions to acquire further details regarding certain parts of the maps. In these interviews, we focused on discussing the ripples in which the stakeholder had been involved. First, we asked participants to report the outcomes that produced the most personal pride, and the most important outcomes of JOGG in the community. Second, we asked participants to share their stories about these outcomes and specific parts of the map in which they were involved. We added the input from the additional interviews to the ripple effects map where necessary.

### Analyses

The REM sessions and the additional interviews resulted in one map per community, which comprised the merged maps from the two REM sessions with further details from the additional interviews. IH coded the transcripts from the REM sessions and interviews according to the completed maps, using the MAXmaps function in MAXqda, which enables graphic data representation. The result of this coding phase was a tidy map with underlying data segments for each community. These maps formed the starting point for analysis.

Following recommendations from Nobles et al. (2022) and Peterson et al. (2019), data analysis was an inductive and iterative process. First, IH conducted a thematic analysis of the outcomes and long-term impact of JOGG as perceived by community stakeholders to acquire insight into the types of outcomes that participants associated with the JOGG approach. Second, we focused the analyses on causal pathways, following the guidelines of Nobles et al. (2022) to identify and analyse “impact pathways”: chains of actions and outcomes within the REM maps. For each community, three members of the research team (IH, MH, DC) identified these pathways to long-term impacts and subsequently analysed how these long-term impacts were connected to other outcomes and JOGG actions to illustrate how the programme works [28, 29].

Third, we conducted a cross-case analysis [35, 36] to identify common patterns and to explain differences between communities. IH, MH and DC used the maps and identified impact pathways from the five communities to jointly analyse and discuss (1) how outcomes related to each other to support each type of long-term impact identified by the thematic analysis in the first step, (2) JOGG’s role in accomplishing outcomes and impact, and (3) feedback loops in the process. During analyses and discussion, we iteratively compared the cases, gradually generating overarching causal pathways for each long-term impact. By moving to a higher level of abstraction, the final overarching pathways were applicable for all communities.

## Results

The results section first describes local stakeholders’ perceptions regarding JOGG’s outcomes and long-term impact. Then, we present causal pathways to these long-term impacts and we explain key mechanisms through which JOGG contributed to these pathways.

### Outcomes and long-term impact of the JOGG approach

Table 2 summarizes the outcomes and long-term impacts that participants perceived to be associated with the JOGG approach in their community. Outcomes that participants believed to be direct outcomes of the JOGG approach were divided into three themes: (a) the commitment of organizations, (b) the implementation of activities that were developed in collaboration with the local JOGG team and (c) changes in children and parents. Participants for example noted that children were more knowledgeable about healthy nutrition or drinking more water than they had been:

What we constantly emphasize is, ‘guys, if you have a moment of rest or anything, don’t grab cola or chips’. That was done initially, but now, there’s just a water tap about a metre or two away, and it's being used a lot. When we have a break, they all line up and drink from the water tap. (sport service C3)

**Table 2:** Summary of JOGG outcomes and long-term impacts according to participants

Themes	Identified outcomes
Stakeholder commitment (public and private)	• Familiarity with JOGG • Support of JOGG’s goals • Awareness of their role in promoting healthy lifestyles • Knowledgeable about health and why promoting healthy lifestyles is important • Motivation and enthusiasm to participate in JOGG • Willingness to promote healthy lifestyles
Implementation of actions to promote healthy lifestyles	• Implementation of activities to promote knowledge and awareness about physical activity and healthy nutrition in children and parents • Implementation of activities to promote physical activity and motor skills • Implementation of interventions aimed at overweight children
Individual-level outcomes	• Increased awareness and knowledge among parents and children about a healthy lifestyle • Healthier food and drink choices and increased participation during organized neighbourhood events for children • Increased water consumption among children

Themes	Identified long-term impacts
Intersectoral collaboration for promoting healthy lifestyles	• Increased awareness of and contact with other organizations regarding health promotion • Reduced overlap between similar organizations • New collaborative projects that promote a healthy lifestyle • Strengthening existing projects (for example, by better connecting different projects)
Stakeholder ownership for promoting healthy lifestyles	• Stakeholders’ knowledge of their status as role models • Stakeholders’ integration of healthy lifestyles into their daily work • Stakeholders’ active encouragement towards healthy lifestyles across the community
Policies that promote healthy lifestyles	• Policy changes in schools (for example, about drinking water at school, fruit snacks, birthday treats) • Policy changes in childcare centres and preschools (for example, lunch meals, outdoor play, fruit snacks) • Policy changes in sport clubs (for example, changes to the canteen, drinking water, healthy snacks at events) • Healthy lifestyle activities embedded in organizational routines • Resources for promoting healthy lifestyles integrated in municipal health policy
Change in social norms regarding the promotion of healthy lifestyles	• Increasing numbers of organizations promoting healthy lifestyles • Normalizing responsibility to promote healthy lifestyles

Participants also described numerous long-term impacts; sustainable changes that manifested over several years and to which they believed JOGG had contributed. These long-term impacts comprised four themes. First, stakeholders associated JOGG with strengthened intersectoral collaborations to promote healthy lifestyles, especially between public organizations in the community (for example, youth work and public health service). Second, according to participants, JOGG had contributed to a level of ownership for promoting healthy lifestyles in relevant community organizations. Third, participants associated JOGG with developed policies both at the municipal level and within organizations to promote health, such as school nutrition policies. Fourth, participants noted that JOGG had contributed to a change in social norms regarding the promotion of healthy lifestyles. Participants explained that some health-promotion actions had become routine within their organization or within the community overall and that an increasing number of organizations now assumed responsibility for promoting healthy lifestyles:

In practice, we really see that drinking water, eating vegetables, it’s becoming more of a norm, especially at events and sports days. But also, at children’s healthcare centres it is a more discussed topic. More and more childcare organizations and schools are getting involved. (health service C1)

I remember when we had just started with JOGG. We organized a school soccer tournament, and they were distributing lemonade and chips and all kinds of unhealthy stuff. That is unthinkable now. (school C2)

In none of the five communities participants had mentioned negative outcomes of the JOGG approach. However, they did reflect on areas for improvement. For example, participants expressed doubts about whether the actions implemented by stakeholders effectively reached the children who needed it the most.

### Causal pathways

The ripple effects maps revealed how different outcomes rippled into long-term impacts, thus demonstrating the various series of smaller steps that are needed to create impact. We describe the causal pathways – the chains of interacting actions and outcomes – of the four long-term impacts: policy changes, stakeholder ownership, intersectoral collaboration and social norm changes regarding the promotion of healthy lifestyles.

#### Causal pathway to implement policies that promote healthy lifestyles within stakeholder organizations

**Fig. 1:**
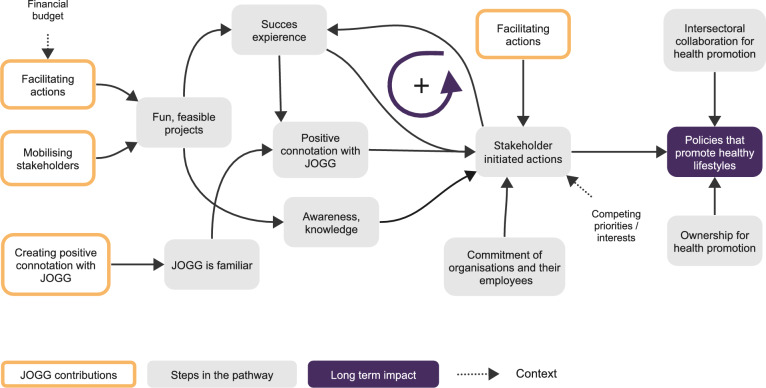
Causal pathway to implement policies that promote healthy lifestyles

Figure 1 illustrates the pathway to policies within stakeholder organizations that promote healthy lifestyles. First, JOGG teams mobilized stakeholders by establishing initial contact and lobbying for participation in the JOGG approach or promotion of healthy lifestyles. Participants described that this contact had been essential to encourage their involvement in the beginning:

In the beginning, when JOGG was still small, the coordinator physically went to all the schools. So, I regularly had conversations with the coordinator. Just that aspect of awareness, even within ourselves, because we have to convey it to those children. So yeah, lobbying in the beginning and investing that energy in all those conversations, I think that has been very intense, but I think it has certainly been very good. (school C5)

Additionally, JOGG teams created a positive connotation with JOGG among relevant local stakeholders by, for example, being visibly present at events or communicating through social media. The increased familiarity with JOGG and positive connotation that this created furthered the JOGG team’s efforts in establishing initial contact with stakeholders.

Second, after stakeholders were mobilized, the JOGG team facilitated a small project at the stakeholder organization that were usually feasible and fun, a water-drinking day or a running event for example, so that it was easy for stakeholders to participate. Although such small and incidental projects alone would not immediately change children’s lifestyles, they catalysed further actions by creating stakeholders’ knowledge and awareness about healthy lifestyles and formed a stepping stone to continue their efforts regarding upcoming projects. A participant described this process as follows:

I think that JOGG is able to create an accessible entry point where the school starts to think and becomes aware that there’s more to education than just teaching math and Dutch. They consider practical ways for the school to contribute to the health of the children. Drinking water is super tangible, very accessible and actually very easy to implement. This brings about an awareness within the school to further explore how can we do things outside the regular curriculum for the well-being and health of the children. (health service C2)

Third, organizations continued their efforts to promote healthy lifestyles. This ranged from participating in another project facilitated by JOGG to initiating projects themselves. An important condition for continuing efforts was organizational commitment to participate in JOGG and promote healthy lifestyles. Additionally, successful experiences were an essential mediator to continue efforts. Achieving successes created a positive connotation with JOGG and the benefits of healthy lifestyles. For example, one participant explained that their school now pays the costs to sustain the project after first experiencing the benefits:

I’m now using that lifestyle coach again, but the school now has seen that it benefits us, so now we also pay her. [...] When the school notices that hey, this works, or we really benefit from this, you see that the school also allocates budget for it. Whereas if that hadn’t been the case, if I had approached them saying, ‘Oh, there is a lifestyle coach who wants to work with us, but it costs so much’, then the school would have said, ‘We don’t have space for that now; maybe next year’. (school C3)

Successful experiences initiated and reinforced a feedback loop, thereby increasing stakeholder motivation and commitment to participate in new projects, which created new successful experiences. To attain success, proper project facilitation by JOGG was essential because the goal needed to suit stakeholders’ needs and routines, and budgets were needed to organise the project.

Fourth, after multiple successful experiences, stakeholders moved from incidental projects towards organisational policy changes. This was described as a slow process that comprised small steps. For example, one participant stated the following:

That is gradual, you know. Thinking like, okay, first, let’s focus on water, and then afterward, we’ll look at making the sandwiches a bit healthier too, instead of just white bread with chocolate spread. So, I think that’s really the next step we need to start working on. (childcare C3)

Policy changes were facilitated by intersectoral collaborations and organisational ownership. Intersectoral collaborations provided the means to implement policies; for example, sport organisations could structurally organise physical activities at school, whereas organisational ownership for promoting healthy lifestyles was essential to implementing policy changes. As one stakeholder stated,

If you want to move away from juice boxes and transition to drinking water, it takes a few years; you cannot suddenly say, ‘Let’s change’. Teachers also need to understand why that is important, just like eating fruit or physically active learning, they just need to experience it. (school C4)

#### Causal pathway to encourage stakeholder ownership for promoting healthy lifestyles

**Fig. 2:**
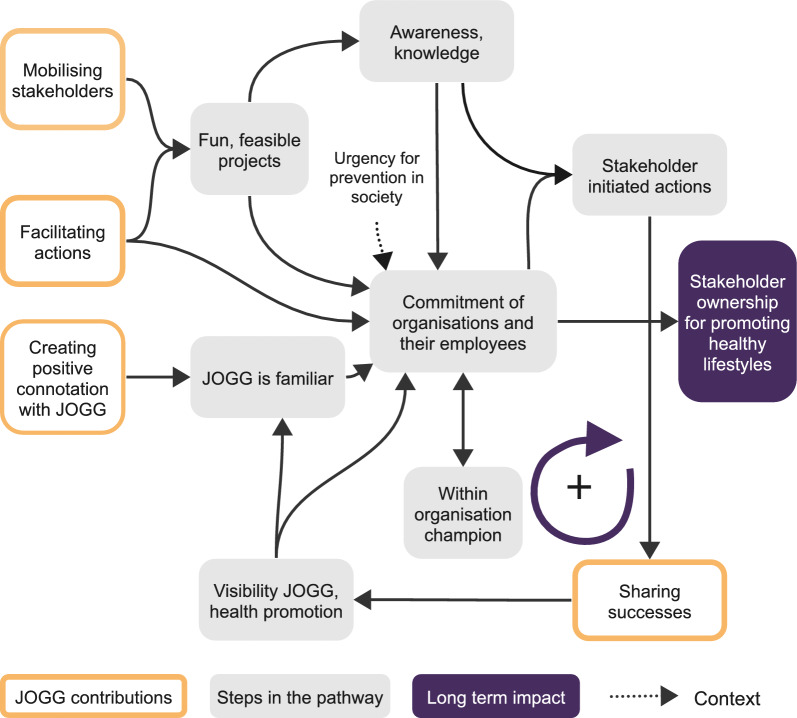
Causal pathway to encourage stakeholder ownership for promoting healthy lifestyles

Figure 2 portrays the pathway to stakeholder ownership for promoting healthy lifestyles in children and youth. The first step in this pathway was the development of stakeholder awareness and knowledge, stimulated by the fun and feasible projects that JOGG facilitated. General knowledge and awareness of healthy lifestyles combined with knowledge about children’s healthy lifestyles as well as familiarity with JOGG and their goals resulted in stakeholder commitment to participate in the JOGG approach. Several participants noticed that organizational participation in JOGG had additionally increased over the years because of a growing urgency for prevention in society and national policy:

Look, 15 years ago you really did not stand a chance with sport clubs to implement a healthy sport canteen. […]. They would have kept the door closed back then. But now, given the developments in society, they open the door. (sport service C4)

Second, after commitment from one or several individuals within a stakeholder organization, these stakeholders became champions who spread awareness within their organizations to achieve the commitment of all employees involved:

At one point, there was a teacher who was interested in the theme. When she started getting involved and the principal eventually began giving her space to work with JOGG, that’s when things really started taking off. She became the contact person for the school; she also initiated various initiatives and implemented them. (sport service C4)

Third, JOGG teams actively created visibility of stakeholders’ actions to promote healthy lifestyles by sharing successes, thereby catalysing a reinforcing feedback loop. For example, they celebrated the opening of a healthy canteen or water tap with an official visit from a local politician, and they published stories about initiatives in local newspapers. The visibility of JOGG and stakeholders’ actions to promote healthy lifestyles stimulated other stakeholders to participate. Different stakeholders stated that they learnt about other stakeholders’ actions through newsletters or social media, which normalized engagement in promoting a healthy lifestyle. As one participant related,

Those newsletters are really comprehensive; they contain everything they do. For instance, if a school achieves a certificate, they mention that. Recently, in November, it was about the drinking water lessons, and they also mentioned that we did those lessons, you know. [...] You can also see it happening in other schools and even in secondary education. So, I have a feeling that it’s becoming more and more normal, so we can try to achieve that within our school as well. (school C2)

The more stakeholders were committed to and participated in promoting children’s healthy lifestyles, the more visibility they received, and the more other stakeholders became aware of their role in promoting healthy lifestyles.

Fourth, stakeholders’ commitment and awareness of their role in promoting children’s healthy lifestyles eventually evolved into ownership, which meant that employees within the stakeholder organizations actively assumed responsibility to promote healthy lifestyles. One participant described the following:

Without me specifically asking for it, all the treats that were made during the Saint Maarten event were healthy. So, at that point, I thought, ‘Okay, I do not need to do anything anymore’. (school C3)

Similar to implementing policy changes, the pathway to ownership for promoting healthy lifestyles was described as a gradual development over the years.

#### Causal pathway to foster intersectoral collaboration for promoting healthy lifestyles

**Fig. 3:**
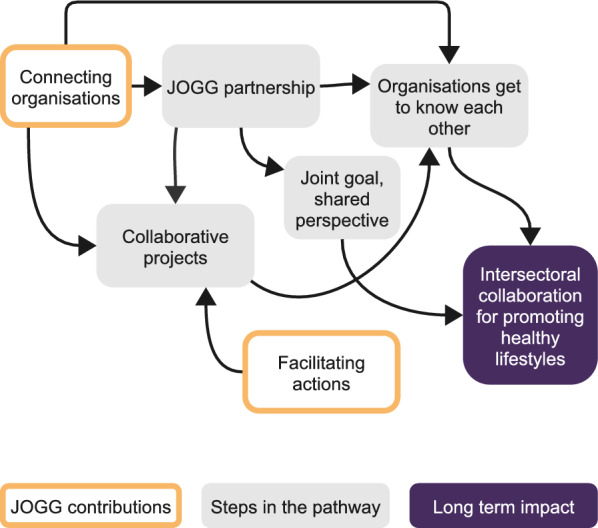
Causal pathway to foster intersectoral collaboration for promoting healthy lifestyles

The third causal pathway that we identified was the pathway to intersectoral collaboration for promoting healthy lifestyles (see Fig. 3). This pathway was initially enacted when the JOGG team connected different projects and organizations, either directly or by involving various stakeholders in a collaborative project. The JOGG team then facilitated such collaborative projects by providing materials, budget and expertise or by managing the project to ensure smooth collaboration. Another way they united organizations was by forming partnerships in which different stakeholders were involved and collaborated. This provided joint goals through a mission that connected the stakeholders, which heightened their motivation to promote healthy lifestyles and stimulated their collaboration. For example, one participant described a partnership meeting:

Yeah, that was really a moment when we thought, ‘Wow, that’s really cool’. The councillors were there, and all the policy-makers were there, and everyone said, ‘Yes, this is really what we find important’. I went home feeling inspired, thinking, ‘Wow, we’re all in this together’. (youth work C5)

Participants explained that the most important result of connecting with other stakeholders was that they became aware of each other and each other’s work. This in turn strengthened intersectoral collaboration, leading to new initiatives:

We easily contact each other and thereby organize activities. For example, the toddler evening walk event. We as a childcare organization sponsor it, the sport service organizes it, the supermarket is also involved. That is how we get a lot of children active. [….] and that connection between the organizers has already been established in the past [by JOGG], and we are now making use of it. JOGG is not involved in this activity itself. (childcare C2)

Intersectoral collaboration, in turn, facilitated the implementation of health promotion actions within the individual organizations. For example, structural intersectoral collaboration between a supermarket and a sport organization enabled the sport organization to provide fruit during their activities.

#### Combined causal pathway to change social norms towards promoting healthy lifestyles

Jointly, ownership, policy changes and intersectoral collaboration contributed to a healthy lifestyle becoming the social norm. Participants described that the social norms changed because numerous community stakeholders assumed ownership for promoting children’s healthy lifestyles and embedded this in their policies. The more stakeholders demonstrated ownership and changed policies, the easier and more natural it became for others to participate as well:

When I look back at a few years ago, schools and sometimes parents were hesitant to say, ‘Yes, my child has to drink water’. They found that very difficult. Well, now, if they know that it is also happening at school, you just hear them say, ‘Yes, at school you have to, so we do that here too’. That broadening, that normalization, really helped. (health service C2)

Participants additionally recognized that policy changes needed to be implemented across numerous organizations in different sectors to impact health:

At [an after-school programme], they have really strict policies regarding healthy nutrition. But we all need to do it together, right? So, it is great that the after-school programme does not give too many unhealthy things, but if then, if we at 5 minutes before school ends, we give something like an ice cream with 299 calories, then we are not doing it right. (school C5)

#### Key mechanisms of the JOGG approach

**Fig. 4:**
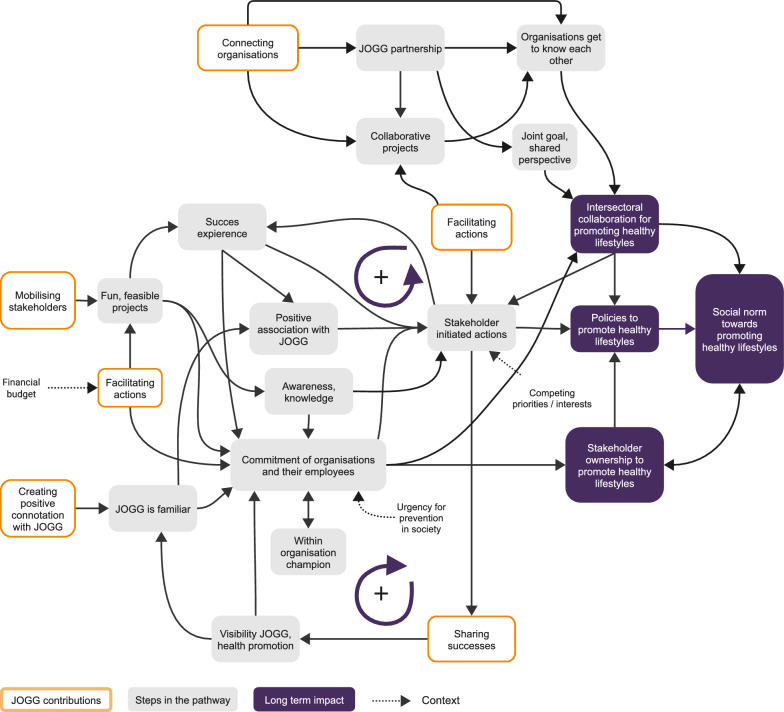
Causal pathway to change social norms towards promoting healthy lifestyles

Figure 4 portrays how the causal pathways related to each other and the key mechanisms through which JOGG contributed to these pathways. We identified five key mechanisms: connecting organizations, facilitating actions, creating a positive connotation with JOGG, mobilizing stakeholders and sharing successes. These contributions are the key mechanisms that activate the impact pathways and enable the success of the JOGG approach. We found that all key mechanisms were present in all communities. However, JOGG teams varied in the mechanisms on which they focused, as these were dependent on the community possibilities and the team skills.

Actions that were implemented by the JOGG team to achieve each key mechanism varied. Table 3 presents the types of actions that were conducted for each mechanism. For example, creating a positive connotation with JOGG was achieved using communication materials (for example, social media), organizing a community-wide campaign or by being visibly present at events or activities. Some actions achieved multiple key mechanisms; for example, by organizing stakeholder meetings, the JOGG team connected organizations and mobilized them for action.

**Table 3:** Summary of key mechanisms and types of action for each key mechanism

Key mechanism	Implemented actions
Connecting organizations	• Contacting and connecting stakeholders personally • Maintaining an overview of community initiatives and stakeholders • Coordinating working group meetings with organizations • Being involved in other health initiatives • Organizing partnership meetings • Organizing a health week to which stakeholders contribute • Promoting healthy lifestyles through ambassadors within one’s own organization
Facilitating action	• Initiating collaborative projects • Managing projects • Providing resources (materials, finances or sponsorships) • Providing expertise • Offering evaluations • Communicating about potential resources (personally, through website or concrete offers for schools)
Creating positive connotations with JOGG	• Sharing activities and successes through fact sheets, newsletters, and social media • Organizing partnership meetings • Organizing campaigns • Organizing a health week to which stakeholders can contribute • Being visibly present at events by, for example, distributing fruit and water • Promoting JOGG activities through ambassadors within one’s own organization
Mobilizing stakeholders	• Contacting stakeholders personally • Organizing partnership meetings • Initiating collaborative projects • Celebrating and sharing successes • Conducting progress and needs meetings with schools • Organizing campaigns • Organizing a health week to which stakeholders can contribute
Sharing successes	• Promoting the achievement of certificates or badges that are provided by the national JOGG organization or other national organizations (for example, national food centre or healthy school canteens) • Celebrating successes by, for example, organizing a party or event at the opening of a new water tap • Sharing activities and successes through fact sheets, newsletters and social media • Using partnership meetings to allow stakeholders to promote their successes

## Discussion

In this study, we used REM to explore the outcomes, long-term impact and causal pathways in a community-based programme for child health promotion and obesity prevention in the Netherlands (the JOGG approach). Local stakeholders perceived JOGG to contribute to four types of long-term impacts: (1) new policies that promote healthy lifestyles in organizations and local government, (2) increased ownership of healthy lifestyles among stakeholders, (3) improved intersectoral collaboration and (4) increased positive social norms among stakeholders towards the promotion of healthy lifestyles. The causal pathway to these impacts comprised small initial outcomes, such as stakeholders supporting JOGG’s goals and participating in feasible projects. These created preconditions, for example, stakeholders’ awareness and knowledge of their role in health promotion, that eventually enabled the achievement of long-term impact.

Although the exact actions that were implemented varied widely between communities, the underlying working mechanisms were similar. We identified five key working mechanisms through which JOGG activated and catalysed the process towards long-term impact across all five communities. Mechanisms that prompted initial participation in the JOGG approach included (1) creating a positive connotation with JOGG so that stakeholders were familiar and willing to participate, (2) mobilizing stakeholders for participation, (3) facilitating projects to promote knowledge and awareness in stakeholders. After stakeholders were involved, it remained important to facilitate projects to enable successful experiences with promoting healthy lifestyles, which in turn increased willingness to do more. Other mechanisms that catalysed the process towards long-term impact included (4) connecting stakeholders to stimulate the intersectoral collaboration that is needed to effectively promote healthy lifestyles and (5) sharing successes to make stakeholders’ activities visible, which gradually created a norm of stakeholder participation and ownership to promote healthy lifestyles.

We found REM [27, 28] to be a useful method to acquire an understanding of how the JOGG approach works. First, the participatory mind-mapping approach was useful to gain insight into participants’ experiences, as it allowed participants to share their stories and reflect on outcomes and barriers that were important to them, while concurrently and systematically discussing the JOGG approach as well as community health promotion. The element of appreciative inquiry created a positive energy in the sessions, especially with participants who had been closely involved with the JOGG approach since it allowed them to see how their efforts gradually unfolded.

Second, REM offered a systematic way to capture the various outcomes of the JOGG approach, including those that emerged over time. Outcomes of community-based programmes can vary significantly across communities [10]. REM provided a broad picture of potential outcomes and impacts, which we would not have been able to capture when studying a predefined set of outcomes. For instance, while we observed policies that promoted healthy lifestyles across all communities, the nature of these policy changes varied widely. In one community, physical activity interventions were developed and structurally implemented by schools, whereas another community focused on water-drinking policies in childcare centres, and yet another community integrated annual lessons to promote cycling to school.

More importantly, REM allowed us to improve our understanding of JOGG’s working mechanisms. By capturing interactions between different outcomes and actions of the implementing team, REM provided insight into how JOGG contributed to the complex processes towards impact. We were thereby able to investigate how the JOGG approach stimulated changes to the system driving obesity by, for example, propelling feedback loops and implementing mutually reinforcing actions, rather than assessing how one action led to one specific outcome. Consequently, the working mechanisms that we identify in this study extend beyond generalized best practices [11, 13], barriers and facilitators to implementation [37] that have been identified in previous process evaluations of community-based obesity prevention programmes. The working mechanisms that we identify can inform future developments in the JOGG approach and other community-based obesity prevention programmes [10] to improve, for example, guidelines for local implementation.

In our study, we added a new step to the REM output analysis to identify JOGG’s key mechanisms across contexts. We first followed the approach of Nobles et al. [28] for identifying and analysing causal pathways to impact. Then, we studied the REM output across communities to compare impact pathways, using guidelines for comparative case study research [35, 36], which enabled us to move the identified causal pathways to higher levels of abstraction and form more generalizable theories about how the JOGG approach works. This significantly improved our understanding of key mechanisms in the JOGG approach. Although the types of actions implemented in different communities varied, we believe that the function of these actions in the causal pathways was similar, which aligns with suggestions that complex programmes define the function of key programme elements instead of the specifying the precise form in which the programme elements should be implemented [25, 38].

### Strengths and limitations

This study has several limitations. First, most of the REM session participants were initially recruited by the local JOGG team, which may have introduced selection bias. Participants who had more negative or neutral experiences may have been less prone to participate and therefore underrepresented, which may have led to an overestimation of JOGG’s positive outcomes. We attempted to mitigate these biases by asking participants to critically reflect on JOGG’s contributions and potentially negative consequences. Additionally, we deliberately did not include the local JOGG team in the sessions so that we could capture the perspective outside of the programme implementers.

REM sessions were conducted online, partially due to COVID-19 regulations, which was both a strength and a limitation. Online sessions better accommodated potential participants’ schedules, which improved their willingness to participate. Therefore, we may have been able to speak to more participants than would have been possible if we had conducted in-person sessions. However, as previous scholars note [39], stimulating interaction and discussion between participants is more challenging online. To facilitate these discussions, we conducted shorter REM sessions in smaller groups. Consequently, the ripple effects maps were generated in different subgroups and later merged by the researchers. We found that maps from different sessions often complemented each other, and we asked participants to verify the merged maps; however, this process may still have elicited different results than one large REM session in which participants jointly work towards a shared understanding [39].

Finally, the retrospective design allowed us to acquire insights into the long-term impacts of the JOGG approach and the processes that enabled these impacts; however, notable limitations of the retrospective design are recall and selection biases [36]. The longer JOGG had been implemented in a community, the more difficult it was for participants to recall events and the fewer participants we found who had been involved since the beginning. Consequently, JOGG’s outcomes and contributions in the process may have been overestimated. As proposed by Nobles et al. [28], REM sessions are ideally conducted multiple times over the years, which not only prevents recall and selection biases but also allows researchers to gain significantly more detailed insights into the process and aspects of the programme that did not lead to outcomes.

### Implications for practice and future research

The results of this study provide a new way of understanding successful implementation of the JOGG approach. Rather than focusing on the exact actions that should be implemented, future JOGG teams should consider how their actions begin the process towards long-term community impact. The five key mechanisms identified in this study can be used by the national JOGG organization to support successful local implementation. Since these mechanisms are defined on an abstract level of their function, they are applicable in diverse contexts. Thereby, the key mechanisms may inform future implementation of the JOGG approach as well as other community-based programmes for health promotion. Furthermore, the causal pathways towards the long-term impacts described in this study may be used to guide JOGG teams to better understand which small steps are needed to achieve long-term impact and to identify which steps are needed in the current situation.

Moreover, the results of this study provide guidance for monitoring and evaluation. The extent to which the key mechanisms are realized may be indicators for successful implementation. The outcomes that are identified in this study could be monitored to examine the effects of the JOGG approach, which could be supplemented with an examination of emergent outcomes. Notably, few participants associated the JOGG approach in their community with an outcome related to the overarching goals of the JOGG approach: healthier lifestyles and the prevention of overweight, although previous effect evaluations have focused on such outcomes [40, 41]. It remains to be determined whether such outcomes can be expected from the current programme and whether these are appropriate indicators for the programme’s effects.

In this study, we identified potential outcomes and key mechanisms through qualitative methods. Future researchers could use mixed-methods designs to strengthen the evidence by validating the key mechanisms and outcomes in different contexts [42, 43]. Qualitative studies can further unpack the key mechanisms and causal pathways that are proposed in the current study, while quantitative studies can validate the outcomes and key mechanisms across contexts by, for example, systematically monitoring the implementation of key mechanisms and potential policy changes in a larger number of communities.

## Conclusions

The long-term impacts of the JOGG approach include stakeholder ownership, policy changes, intersectoral collaboration and a change in social norms towards promoting healthy lifestyles. REM is a useful method to acquire insight into how the JOGG approach works, as it uncovers interactions between different outcomes and long-term impact as well as the contribution of the JOGG approach to this process. By applying REM in five communities, we obtained insight into the causal pathways to impact and key mechanisms across contexts.

## Supplementary Information

Additional file 1.

Additional file 2.

## Data Availability

The data generated and analysed during the current study are not publicly available due to content that could compromise research participant privacy but are available from the corresponding author on reasonable request.
