# Supplementary material for: Uncovering the key working mechanisms of a complex community-based obesity prevention programme in the Netherlands using ripple effects mapping
Source: Health Res Policy Syst. 2024 Sep 4;22:122. doi: 10.1186/s12961-024-01182-y (PMC11373344; doi:10.1186/s12961-024-01182-y)
Supplement: Supplementary file 1 — Additional file 1. [file 12961_2024_1182_MOESM1_ESM.pdf]

**Supplementary file 1 – COREQ checklist**

**Domain 1 research team and reflexivity**

1. *Interviewer/facilitator*

Irma Huiberts

2. *Credentials*

MSc.

3. *Occupation*

Researcher at the Mulier Institute (scientific sport research institute; https://www.mulierinstituut.nl/english-about-us-mi/) and PhD candidate at AmsterdamUMC

4. *Gender*

Female

5. *Experience and training*

Two years of qualitative research experience and qualitative research training through courses.

*Relationship with participants*

6. *Relationship established*

No relationship existed before the study commencement with any of the research participants. The researcher had only been in contact with the participants to plan the interviews/REM sessions over the phone or email.

7. *Participants knowledge of the interviewer*

The participants were informed about the goal of the interview/REM session, and were informed about the larger study that the interview was one part of. Participants were also informed about the role of the interviewer in the study (lead researcher/PhD candidate on this study) and the funding of the study (by JOGG). Anonymity was ensured in the informed consent and emphasised at the beginning of the interviews.

8. *Interviewer characteristics*

The interviewer had been involved with research on the JOGG approach for two years and had previously conducted interviews with local implementing team and members of the national organisation. Before that, the interviewer was not involved in the topic of health promotion.

**Domain 2 study design**

*Theoretical framework*

*9. Methodological orientation and theory*

In this study we applied a combination of thematic analysis (to analyse the Ripple Effects mapping results) and theory building, gaining inspiration from comparative case study research and grounded theory. More detail is described in the ‘analysis’ subsection of the methods section.

*Participant selection*

*10. Sampling*

Participants were purposefully sampled for their engagement in or being affected by the JOGG approach in the community.

- The research team identified people who had been involved in the programme in documents about the local programme (e.g. programme plans, policy plans, meeting notes, logbooks)
- The local implementation team was asked to suggest people who had been involved in or affected by the programme
- Participants in the REM sessions and interviews were asked to provide suggestions on other people who had been involved in or affected by the programme.

*11. Method of approach*

Participants were initially contacted through email. This email provided an overview of the study, purpose of the REM session/interviews and an explanation of why they were invited to participate. People received a reminder email after not responding within two weeks. People who were interested to participate were sent an informed consent form and were emailed/called to schedule the interview.

*12. Sample size*

Sample size for each of the five communities is reported in Table 1. In total we involved 26 participants in eight REM sessions and conducted additional individual interviews with 24 stakeholders. In each community sampling continued until no new potential participants were suggested by the implementing team or participants in REM sessions/interviews. To facilitate discussion in the online REM sessions, these were conducted in small groups of 3-5 participants.

*13. Non-participation*

Four people declined participation due to a lack of time. In addition to having a lack of time, one of these people also stated to be unsure why they were invited, as they knew very little the JOGG approach in the community.

*Setting*

*14. Setting of data collection*

All REM sessions and interviews were conducted online through video call software: Microsoft teams.

*15. Presence of non-participants*

No. Only the participants and interviewer were present during the interviews. During the REM sessions there was also a research assistant present (MH).

*16. Description of sample*

Participants included professionals from schools, childcare, youth work, health service organisations, sport service organisations, private organisations (e.g., supermarket) and policymakers.

*Data collection*

*17. Interview guide*

The interview protocols can be found in Supplementary file 2. Protocols were developed by IH in collaboration with the research team, based on REM guidelines. The interviews and REM sessions were semi-structured, allowing participants to openly discuss their perspectives and to follow up an areas of interests. During the interviews, participants were asked which outcomes they perceived of the JOGG approach. These outcomes were also used to probe for further information.

After each session the interviewer reflected on the interview and refined the protocol if necessary. We made the following adaptions:

- We added further differentiation in the interview protocol. For those participants who were substantially involved in the JOGG approach, we followed the theming and rippling method to gain insight into the broadness of all JOGG outcomes. For those participants who had been involved in only one project, we assumed an in-depth approach, asking them to share their stories and outcomes to create more detailed descriptions.
- We changed how we presented the miro board to participants. At first we did not include the layers for direct and long-term outcomes, but only presented the middle spot with the municipality name on it. We found that adding the layers

helped participants in organising the outcomes and discussing whether outcomes were direct or indirect.
- During the first REM sessions we provided participants with examples of what types of outcomes they could think of when considering outcomes of JOGG to prompt them to think broadly about outcomes (e.g. projects that were developed with JOGG, resources that were created by JOGG, enthusiasm that was created). However, we during the first to REM sessions we found that participants already had broad perspective on different types of outcomes that could be expected and we decided to not provide examples to avoid steering participants in a certain direction.

The protocol in supplementary file 2 presents the version of the protocol after these adaptions were made.

*18. Repeat interviews*

No repeat interviews were carried out.

*19. Audio/visual recording*

All REM sessions and interviews were audio recorded.

*20. Field notes*

Notes were made by the interviewer, to help the interviewer in the line of inquiry. For example to revisit topics later. After the REM sessions the interviewer made notes to reflect on what went well and what could be improved in for future sessions. The notes were not used in analysis.

*21. Duration*

REM sessions lasted between 75 and 90 minutes. Interview duration varied between 15 and 45 minutes (approximately 30 minutes).

*22. Data saturation*

In each community sampling continued until no new potential participants were suggested by the implementing team or participants in REM sessions/interviews. We believe that for each community we reached a large enough sample size, with different (types of) stakeholders, to gain a detailed account of the JOGG approach in the community from different perspectives.

*23. Transcripts returned*

No. Participants did receive the completed REM map for member check. In addition, all participants received the community specific small report that described the results of the case study for that specific community.

**Domain 3 analysis and findings**

*Data analysis*

*24. Number of data coders*

IH coded the transcripts and interviews. During the identification and cross case analysis of impact pathways, codes were discussed in the research team (IH, MH and DC). In some cases this resulted in jointly checking and discussing underlying data segments.

*25. Description of the coding tree*

Data was coded according to the REM map that was constructed during the sessions, using the MAXmaps function in MAXqda, which enables graphic data representation. When a topic from the additional interviews was not included in the map yet, IH added the additional information to the map.

*26. Derivation of themes*

In the analysis we focussed on the types of outcomes that were reported and impact pathways. The themes that were found within the outcomes and impact pathways were derived from the data.

*27. Software*

MAXqda

*28. Participant checking*

We reported the findings for each community in a community specific small report that described the results of the case study. This was sent to all participants to ensure that it resonated with their experiences. Study findings from results across cases will be published in April 2024 and will be reported back to the programme implementation teams.

*Reporting*

*29. Quotations presented*

Yes, from multiple participants from different communities and organisations. Quotations are identified through community number and organisation/occupation of the participant.

*30. Data and findings consistent 31. clarity of major themes 32. Clarity of minor themes*

During data analysis, the causal pathways described in the results section were iteratively checked against the community specific REM maps and underlying data segments. Themes and significant differences between communities are described in the results section.
