# Supplementary material for: Uncovering the key working mechanisms of a complex community-based obesity prevention programme in the Netherlands using ripple effects mapping
Source: Health Res Policy Syst. 2024 Sep 4;22:122. doi: 10.1186/s12961-024-01182-y (PMC11373344; doi:10.1186/s12961-024-01182-y)
Supplement: Supplementary file 2 — Additional file 2. [file 12961_2024_1182_MOESM2_ESM.pdf]

Supplementary File 2 - interview protocols

Ripple effects mapping sessions

Preparation

- Email informed consent form to participants before start of the session
- Prepare appreciative inquiry questions to put in the chat
- Prepare Miro board as follows:

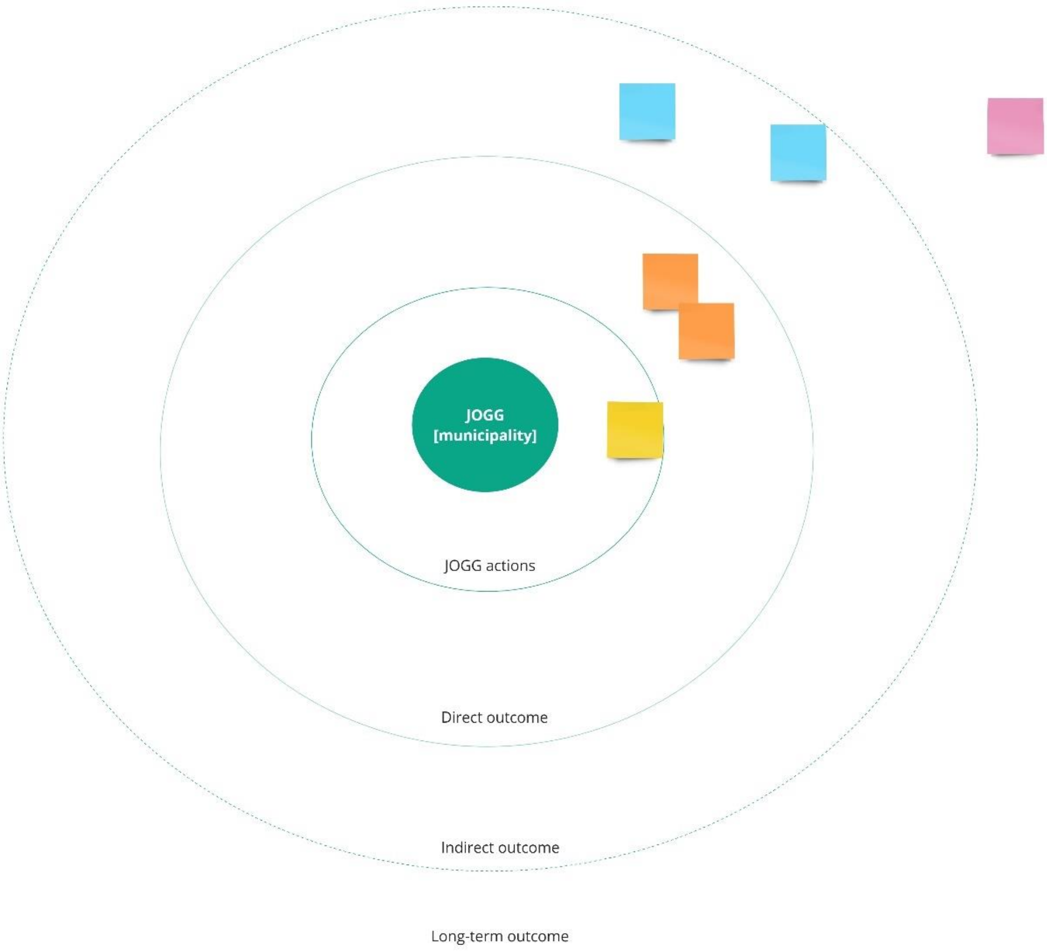

- Ensure access to the miro board for participants
- Facilitator will facilitate the discussion. Research assistant will map the discussion onto the board.

Welcome and introduction (10 minutes)

- Welcome and introduction of the researcher and assistant
- Explanation of the project and the purpose of the meeting: This municipality is part of a larger study on how the JOGG approach works and how it can be successfully implemented under different circumstances. In this study we are interested in the outcomes of the JOGG approach at the

municipal level and want to better understand how this works. Therefore, in several municipalities, we conduct in-depth research, and this municipality is one of them. Today, we want to explore what the JOGG approach has yielded in your municipality. We aim to assess the impact of JOGG, explore the difference it has made in the municipality, and also examine what strengths exist in the current approach, as well as opportunities for improvement. We will do all of this by creating a mind map of the results and impact of JOGG in the municipality. At the end of the session we will reflect on possible improvements and next steps for the JOGG approach in this municipality.

  - You were invited to participate because you have in some way been involved in the JOGG approach in this municipality (in the past)
  - Everyone has their own experience with and perspective on JOGG. We need all of these experiences to get a complete picture. So feel free to share anything that comes to mind. And also give each other space to share their own experiences.
  - We want to get an understanding of the overall impact of JOGG. However, this is not easily distinguished from other developments in the municipality. Therefore, we will also consider broader changes related to lifestyle and health promotion among children and adolescents.
- Questions?
- Introduction of participants
- Check signed consent form
- Start audio recording

Warm-up (15 minutes)

The goal of this part of the session is to gain a first insight into the outcomes of the JOGG approach and to create an interactive and positive atmosphere in the session. If there are more than four participants, this part of the session will be conducted in two breakout rooms, otherwise this will be a group exercise.

- Later, we will make a mindmap of the outcomes of the JOGG approach. Before we do that I want to reflect with you on your experiences of the results of the JOGG approach and how you were involved. Think about the following questions (*put in the chat)*
  - What is the most significant results of JOGG in your organisation? What result are you most proud of?
  - In your opinion, what are the most significant results of JOGG in this municipality overall?
- Ask each participant to share their answers with the group
  - Follow up on whether other participants recognise these results

A research assistant adds the outcomes that are suggested onto the miro board.

Mind mapping (50 minutes)

Instruction: we are going to make a mindmap of the JOGG outcomes and impact in this municipality. We have added everything that has come up so far on the board that I will share with you in a minute. Now we are going to further unpack these results and add on.

- Share miro link. Check if participants are able to open the link and see the board.
- In the middle you can see JOGG. In the circle directly around you can see direct outcomes. Further away we see more indirect outcomes and long-term impacts.
- [research assistant] will add everything we talk about onto the map and you can watch the board and check how it is written down
- Based on this, we can delve deeper into the discussion. We will further elaborate on each of the outcomes: what are examples? Direct or indirect? How are the outcomes connected? Which stories/results are similar?
- About the map:
  - The completed map will provide an understanding of how impact is achieved. How far-reaching is the impact of JOGG? What is the process behind it? What contributes to it?

- Additionally, it will offer an overview of what might still be missing
- This is a draft version. We will tidy it after this session and supplement it with input from others
- Let's create it together. Think along, add to it, be critical. Make sure that what you say is accurately reflected

Mind mapping

Discuss each outcome that was suggested in the first part of the session.

For each more abstract/general outcome:

- Is this a direct outcome, indirect outcome or long-term impact?
- Can you give an example of such outcome? Can you share a story about this? Can you tell us more about that?
- Draw out the ripples by elaborating on each outcome (*use prompts*):
  - What happened to achieve that outcome and how JOGG contributed
  - The ripple effects of that outcome
- Discuss connection to other outcomes

For each specific/concrete outcome

- Is this a direct outcome, indirect outcome or long-term impact?
- Place close to similar outcomes (to form one theme)
- Draw out the ripples by elaborating on each outcome (*use prompts*):
  - What happened to achieve that outcome and how JOGG contributed
  - The ripple effects of that outcome
- Discuss connection to other outcomes
- Cluster similar types of outcomes and ripples

Add new outcomes/impact and discuss
If participants are strongly involved in JOGG and know a lot about the JOGG actions and results, put more focus on adding new outcomes, otherwise focus on in-depth questions to draw out the ripples of the outcomes participants are involved in.

- Other outcomes or impacts of JOGG in the municipality?
- What are other activities or elements of JOGG in the municipality? What are their results?
- What is missing on the mind map?

➔ Discuss each outcome as described above

Probe for negative or unintended outcomes:

- What were negative, perhaps unintended, results of the JOGG approach?
- What disadvantages have you noticed in working with the JOGG approach?

Prompts

Before the outcome

- How was this outcome achieved?
- What contributed to this outcome?
- How did that come about?
- How were you able to achieve that?
- Who was involved?
- How did JOGG contribute?
- What would have happened without JOGG?

Ripple effects

- What happened then?
- What was the consequence?
- What did people start doing differently?
- What did this lead to?
- What changed because of that?
  - For you or your organisation?
  - For children/youth?
- What effect did that have?
- What was the result of that?

Reflection (10 minutes)

In the reflection phase we will have another critical look at the mind map. Have a look at the map as it is now:

- What is most interesting about the mind map?
- Is something missing?
- What was the most significant contribution of JOGG?
- What improvements can be made in the future? Do you see any new opportunities?
- What other stakeholders are relevant to improve our understand of JOGG in this municipality?
    - Who should we speak to?

Wrap up (5 minutes)

- Is there anything that we have not discussed yet that you would have like to have talked about?
- You will receive the completed and tidied mind map to check whether it reflects your ideas
- Discuss how results will be reported
- Thank you for your participation

Additional interviews

Preparation

- Email informed consent form to participants before start of the session
- Prepare stakeholder specific topics in the protocol (based on results REM sessions)
    - Specify the 'elaboration on the mind map' questions
    - If we require more information on a specific part of the mind map from this stakeholder we can spend more time on the part 'elaboration on the mind map' during the interview.
    - Add other topics if necessary
- Depending on the involvement of the stakeholder in JOGG interview will be planned for 0.5 to 1 hour

Introduction

- Welcome and introduction of the researcher
- Explanation of the project and the purpose of the meeting: This municipality participates in a larger study on how the JOGG approach works and how it can be successfully implemented under different circumstances. In this study we are interested in the outcomes of the JOGG approach at the municipal level and want to better understand how this works. Therefore, in several municipalities, we conduct in-depth research, and this municipality is one of them. Today, we want to explore the outcomes of JOGG in your municipality and how JOGG contributed to these outcomes.
- [municipality specific addition: *e.g. we have spoken to several other stakeholders in you municipality and they suggested that we speak to you because….]*
- Questions?
- Check signed consent form
- Start audio recording

Introduction participant and involvement in the JOGG approach

- Introduction participant and organisation
- In what way are you involved in the JOGG approach?
    - Collaborations/role/actions
- How do you experience the JOGG approach?/your collaborations with the JOGG team?

Outcomes of JOGG

<u>Within the organisation</u>

- What is the most significant result of JOGG in your organization? What result are you most proud of?
- How were these outcomes achieved?
    - How did JOGG contribute?

<u>In the municipality</u>

- In your opinion, what are the most significant results of JOGG in this municipality overall?
- For each outcome: draw out the ripples by elaborating on each outcome (*use prompts*):
    - What happened to achieve that outcome and how JOGG contributed
    - The ripple effects of that outcome

Probe for negative or unintended outcomes:

- What were negative, perhaps unintended, results of the JOGG approach?
- What disadvantages have you noticed in working with the JOGG approach?

Future:

- What improvements can be made in the future? Do you see any new opportunities?

Prompts

Before the outcome

- How was this outcome achieved?
- What contributed to this outcome?
- How did that come about?
- How were you able to achieve that?
- Who was involved?
- How did JOGG contribute?
- What would have happened without JOGG?

Ripple effects

- What happened then?
- What was the consequence?
- What did people start doing differently?
- What did this lead to?
- What changed because of that?
  - For you or your organisation?
  - For children/youth?
- What effect did that have?
- What was the result of that?

Elaboration on the mind map

In discussions with other stakeholders in the municipality we found that [*municipality specific results*]. Do you recognise this? Can you tell me more about that?

Municipality specific results are for example:

- *Ripple that this stakeholder is (possible) involved in*
- *Most significant outcomes/ripples from the mind map*

Wrap up

- Is there anything that we have not discussed yet that you would have like to have talked about?
- You will receive the completed and tidied mind map to check whether it reflects your ideas
- Discuss how results will be reported
- Thank you for your participation
